# Co-infections with Chikungunya and Dengue Viruses, Guatemala, 2015

**DOI:** 10.3201/eid2211.161017

**Published:** 2016-11

**Authors:** Thomas Edwards, Leticia del Carmen Castillo Signor, Christopher Williams, Evelin Donis, Luis E. Cuevas, Emily R. Adams

**Affiliations:** Liverpool School of Tropical Medicine, Liverpool, UK (T. Edwards, C. Williams, L.E. Cuevas, E.R. Adams);; Laboratorio Nacional de Salud Guatemala, Villa Nueva, Guatemala (L. del Carmen Castillo Signor, E. Donis)

**Keywords:** dengue, dengue virus, chikungunya, chikungunya viruses, viruses, co-infection, Guatemala

## Abstract

We screened serum samples referred to the national reference laboratory in Guatemala that were positive for chikungunya or dengue viruses in June 2015. Co-infection with both viruses was detected by reverse transcription PCR in 46 (32%) of 144 samples. Specimens should be tested for both arboviruses to detect co-infections.

Chikungunya virus (CHIKV) and dengue virus (DENV) are arboviruses currently circulating in Southeast Asia, Central and West Africa, the Pacific islands, and the Americas, and their transmission can occur simultaneously (*1*). DENV and CHIKV co-infections have been reported from 13 of 98 countries/territories to which the viruses are endemic and are more likely to occur in areas with high transmission intensity. Co-infection rates reported have ranged from 2% in Gabon to 34% in Nigeria (*1*–*3*).

The Americas are currently experiencing an unprecedented number of DENV infections that coincided with emergence of CHIKV infections. A total of 2.3 million DENV infections and 635,000 CHIKV infections were reported in this region in 2015 (*4*,*5*). However, details of the frequency of co-infection are lacking (*1*), although a recent study involving 173 samples from Nicaragua that were positive for either virus found a co-infection rate of 22% (*6*).

Co-infections might be frequently missed by surveillance systems because Pan American Health Organization (PAHO) diagnostic algorithms indicate that DENV-positive samples do not need to be tested for CHIKV or other viruses and vice versa (*7*). The clinical role of co-infection is somewhat disputed because some (*8*) but not all (*1*,*9*) studies reported an association between co-infection and disease severity for symptoms such as diarrhea (*9*) or hemorrhage (*10*).

Although Guatemala has had constant dengue transmission for >20 years (*11*), the first cases of CHIKV infection were reported in September 2014. We report the proportion of patients co-infected with DENV and CHIKV and the association of co-infection with disease severity among patients referred for diagnosis to the National Health Laboratory (NHL) in Guatemala City, Guatemala, in June 2015. Because Zika virus was not introduced until November 2015, we did not test for this virus in this study.

## The Study

Serum samples from febrile persons suspected of having arboviral infections are referred to the NHL in Guatemala for surveillance and confirmation purposes. For logistical reasons, most samples are received from 12 of the 22 districts in Guatemala, and most samples originate from Quetzaltenango, Guatemala, and Escuintla Districts, the largest urban centers in Guatemala.

We selected for screening a convenience set of consecutive samples received in June 2015, which is the peak transmission season for DENV, and reported as having positive results by reverse transcription PCR (RT-PCR) for either virus. Clinical data for patients were obtained from surveillance databases to compare patients with monoinfections or coinfections with DENV or CHIKV. All samples were obtained from patients <5 days of symptom onset. Samples were obtained after patients provided consent and donated to laboratories for further testing. All samples were anonymous. Results of the study were not used for clinical management or surveillance purposes.

During June 2015, a total of 523 samples were tested for CHIKV at the NHL; 328 (63%) were positive for CHIKV RNA by RT-PCR. A total of 514 samples were also tested by RT-PCR for DENV; 75 (9%) were positive (Figure). Seventy-four of samples reported as positive for DENV RNA and 70 samples reported as positive for CHIKV RNA were available for further screening for the other virus.

**Figure:**
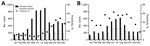
Positivity of samples tested for A) dengue virus and B) chikungunya virus, Guatemala, 2015.

Samples selected were consecutive positive samples from the beginning of the month; there were no additional exclusion or inclusion criteria. We included as many samples as possible within the time available. RNA was extracted by using a Viral RNA Mini Kit (QIAGEN, Manchester, UK) and tested by using the US Centers for Disease Control (CDC; (Atlanta, GA, USA) multiplex DENV RT-PCR and the CDC CHIKV 6856F/6981c/6919-FAM RT-PCR (*12*).

Twenty-five (33.8%) of 74 DENV-positive samples were positive for CHIKV, and 21 (30%) of 70 CHIKV-positive samples were positive for DENV RNA. Co-infection with CHIKV was detected in 4/9 samples containing DENV-1, 40/76 samples containing DENV-2, 0/3 samples containing DENV-3 and 2/7 samples containing DENV-4.

Clinical characteristics of patients with CHIKV and DENV monoinfections and co-infections were similar (Table). Patients co-infected with CHIKV and DENV were more likely to have a rash than those with DENV monoinfections (p = 0.02) and were more likely to be hospitalized than those with CHIKV monoinfections (p = 0.002). All other associations were not statistically significant.

**Table:** Characteristics of patients infected with chikungunya virus, dengue virus, or both, Guatemala, 2015*

Characteristic	CHIKV moninfection	DENV monoinfection	DENV/CHIKV co-infection	p value	
				CHIKV vs co-infection	DENV vs co-infection
No. samples tested	49	49	46		
Sex					
M	15/48 (31)	23/45 (51)	21/46 (46)	0.2	0.6
F	33/48 (69)	22/45 (49)	25/46 (54)	0.2	0.6
Age, y					
<1	2/48 (4.2)	3/45 (7)	5/46 (11)	0.2	0.7
2–16	15/48 (31)	26/45 (58)	19/46 (41)	0.4	0.1
17–30	13/48 (27)	6/45 (13)	9/46 (20)	0.4	0.5
31–60	14/48 (29)	9/45 (20)	10/46 (22)	0.5	1
>60	4/48 (8.3)	1/45 (2)	3/46 (7)	1	0.6
Sign/symptom					
Hemorrhage	7/49 (14)	11/44 (25)	8/44 (18)	0.8	0.6
Arthralgia/myalgia	41/44 (93)	36/42 (86)	35/45 (77)	0.07	0.4
Fever	43/48 (90)	41/42 (98)	46/46 (100)	0.06	0.5
Rash	31/48 (65)	11/43 (26)	23/44 (52)	0.3	0.02
Nausea	26/46 (57)	31/44 (70)	22/44 (50)	0.7	0.08
Vomiting	13/46 (28)	21/43 (49)	16/45 (36)	0.5	0.3
Diarrhea	3/18 (17)	10/34 (29)	6/25 (24)	0.7	0.8
Headache	40/47 (85)	35/42 (83)	40/44 (91)	0.5	0.3
Hospitalization	0/48 (0)	16/45 (36)	8/46 (17)	0.002	0.09
Death	0/48 (0)	4/45 (9)	2/46 (4)	0.2	0.4

*Values are no. positive/no. tested (%) unless otherwise indicated. CHIKV, chikungunya virus; DENV, dengue virus.

## Conclusions

Our findings must be viewed with caution because of the limitations of this study. The study was not powered to analyze clinical associations with a high robustness because of the small number (n = 46) of co-infected samples tested. Also, because samples were selected at a reference laboratory and only symptomatic cases were tested, the prevalence of co-infection might be different in a less selected population.

The PAHO algorithm recommends screening clinical specimens by using consecutive assays and ending screening once a pathogen is identified. CDC in turn recommends simultaneously conducting RT-PCRs for DENV, CHIKV, and Zika virus. Emergence of Zika virus in Latin America has further complicated diagnosis of arboviral infections, and simultaneous co-infections with all 3 arboviruses have been reported (*13*). Although the PAHO approach misses co-infections, the CDC approach is costlier.

Guatemala used the PAHO algorithm because of a shortage of consumable supplies. On further testing, 25 (33.8%) of 74 DENV-positive samples were positive for CHIKV, and 21 (30%) of 70 CHIKV-positive samples were positive for DENV. Because Guatemala reported 49,043 cases of DENV and CHIKV infections in 2015, it is likely that the algorithm missed a large number of co-infections. Patients with single and dual infections had similar clinical manifestations in this limited study.

The frequency of arbovirus coinfections in Guatemala is high. Simultaneous screening for DENV, CHIKV, and Zika virus in disease-endemic areas would improve the quality of arboviral surveillance and potentially aid in clinical management of the disease.
